# Epigenetic, chromatin-associated, and epitranscriptomic regulation in chronic pain: mechanisms and translational prospects

**DOI:** 10.3389/fgene.2026.1904744

**Published:** 2026-09-25

**Authors:** Jianan You, Lei Li

**Affiliations:** The Zhangjiagang Affiliated Hospital of Soochow University, Zhangjiagang, Jiangsu, China

**Keywords:** chromatin remodeling, chronic pain, DNA methylation, epigenetics, histone modification, therapeutic targets

## Abstract

Chronic pain is sustained by durable yet dynamic changes in gene regulation across sensory neurons, spinal circuits, glial and immune cells, and higher brain regions. This review critically evaluates three related but conceptually distinct regulatory layers: chromatin-associated epigenetic mechanisms, including DNA methylation and hydroxymethylation, histone modifications, and chromatin accessibility; non-coding RNAs that directly interface with chromatin-regulatory machinery; and RNA N6-methyladenosine (m6A), an epitranscriptomic mechanism that acts primarily at the RNA level. Across pain models, the most reproducible mechanistic theme is not a uniform increase or decrease in one epigenetic mark, but locus-, cell-, and stage-specific reprogramming that can silence anti-nociceptive genes while activating pro-nociceptive or inflammatory programs. Evidence is strongest where locus-specific perturbation connects a regulatory change to transcription, cellular excitability, synaptic function, and pain behavior; bulk-tissue associations remain mainly hypothesis-generating. Human blood and tissue studies have identified candidate methylation signatures, but none is sufficiently validated for routine diagnosis, prognosis, or treatment selection. Likewise, broad DNMT, HDAC, or histone-modifying interventions show analgesic activity mainly in animals, whereas the negative phase two trial of the HDAC6 inhibitor ricolinostat illustrates the translational gap. We therefore argue that future progress will depend on cell-resolved longitudinal multi-omics, causal epigenome editing, human tissue validation, and biomarker studies designed around prespecified clinical utility rather than statistical association alone.

## 1 Introduction

Chronic pain is generally defined as pain that persists or recurs for longer than 3 months and may become a disease in its own right rather than merely a symptom of continuing tissue injury (Treede et al., 2019). Its causes include nerve injury, inflammation, surgery, diabetes, chemotherapy, cancer, visceral disorders, and post-traumatic conditions. Clinically, chronic pain often combines evoked hypersensitivity or spontaneous pain with sleep disturbance and affective or cognitive comorbidity (Wang and Doan, 2024; Zhang et al., 2025; Yang and Chang, 2019). At the systems level, chronification involves coordinated plasticity in peripheral nociceptors, spinal dorsal horn circuits, glial and immune populations, descending modulatory pathways, and brain networks involved in salience, motivation, emotion, and memory (Zhang et al., 2025; Yang and Chang, 2019; Woolf, 2010; Costigan et al., 2009; Ji et al., 2018; Baliki Marwan and Apkarian, 2015).

Persistent transcriptional changes are central to this plasticity, but their interpretation requires more than a list of differentially expressed genes. Injury-related activity, inflammatory mediators, metabolic state, and psychological stress can alter the regulatory context in which genes are transcribed, thereby stabilizing or constraining cellular states over time (Descalzi et al., 2015; Xu and Xin, 2021; Topham et al., 2020; Buchheit et al., 2012; Jiang W. et al., 2022; Liang et al., 2015; Denk et al., 2012). In chronic pain, these changes can preserve neuronal hyperexcitability, weaken endogenous inhibition, sustain neuroimmune signaling, and reshape the affective meaning of pain. Recent large-scale human multi-omic evidence further supports this systems-level perspective: multisite chronic pain is associated with coordinated biochemical, metabolic, and proteomic signatures that track immune-metabolic-psychiatric disease burden, including rheumatoid arthritis and depression (Wang et al., 2026). These findings place persistent pain within broader neuro-immune-metabolic dysregulation rather than within nociceptive circuits alone. Importantly, the same regulatory enzyme or chemical mark may have opposite consequences at different loci. Hypermethylation can silence an anti-nociceptive potassium channel or opioid receptor, whereas demethylation can activate a pro-excitatory chemokine receptor or neurotrophin. Direction of effect therefore cannot be inferred from the global abundance of a mark alone.

The original literature frequently uses the term “chromatin remodeling” as an umbrella for DNA methylation, histone modifications, chromatin accessibility, non-coding RNAs, and RNA m6A. This usage is broader than the strict molecular definition of chromatin remodeling, which refers most directly to ATP-dependent complexes that reposition, eject, or restructure nucleosomes. To avoid conceptual ambiguity, this review distinguishes chromatin-associated epigenetic regulation from epitranscriptomic regulation and includes ncRNAs only insofar as they interact with chromatin or help integrate chromatin and post-transcriptional control. The aim is not merely to catalogue studies, but to identify convergent principles, weigh causal strength, expose contradictory or context-dependent findings, and assess translational readiness.

## 2 Conceptual framework: regulatory layers and evidence strength

### 2.1 Chromatin-associated epigenetic regulation

Chromatin-associated regulation in this review comprises DNA methylation and TET-dependent hydroxymethylation, covalent histone modifications, chromatin accessibility, enhancer activity, and ATP-dependent nucleosome remodeling. These mechanisms are physically linked to DNA or nucleosomes and can influence transcription factor occupancy, promoter-enhancer communication, and the probability that a gene remains active or repressed. Nevertheless, common shorthand should be interpreted cautiously: histone acetylation is often associated with accessible chromatin, H3K4me3 with active promoters, and H3K27me3 or H3K9me2/3 with repression, but the biological outcome depends on genomic location, reader proteins, cellular identity, and temporal context (Descalzi et al., 2015; Jiang W. et al., 2022; Liang et al., 2015; Denk et al., 2012).

### 2.2 Non-coding RNA and m6A: related but distinct layers

Non-coding RNAs span several mechanistic categories. Some lncRNAs recruit DNA methyltransferases, histone-modifying enzymes, or ATP-dependent remodeling complexes and are therefore directly relevant to chromatin-associated regulation. Other miRNAs, lncRNAs, and circRNAs act predominantly through mRNA degradation, translational control, antisense pairing, or competing-endogenous-RNA networks; these are important to pain biology but are not themselves chromatin remodeling. RNA m6A is even more clearly distinct: it is an epitranscriptomic modification installed on RNA and primarily regulates RNA processing, localization, stability, or translation. It is included here because it can intersect with ncRNA maturation and transcriptional networks, not because it is a chromatin mark.

### 2.3 A hierarchy for interpreting evidence

Evidence should be weighted according to causal resolution. Locus-specific methylation or chromatin perturbation linked to target-gene expression, electrophysiology, and behavior provides stronger mechanistic support than correlations between global mark abundance and pain behavior. Cell-type-specific genetic manipulation is more informative than pharmacological inhibition in bulk tissue, because broad inhibitors can affect multiple enzymes, cell populations, and genomic loci. Human peripheral-blood associations are valuable for biomarker discovery but do not establish that the same change occurs in nociceptive tissue or causes pain. Table 1 summarizes representative studies using this hierarchy.

**TABLE 1:** Representative regulatory mechanisms in chronic pain and their evidentiary limitations.

Regulatory mechanism	Tissue/cell type	Model or specimen	Target/pathway	Functional consequence	Critical interpretation
Genome-wide DNA methylation reprogramming	Bulk DRG	SNL rodents	Distributed CpG sites, largely outside CpG islands	Persistent methylation changes associated with hypersensitivity; methyl donors reduced pain behavior	Shows broad reprogramming, but bulk DRG cannot assign changes to neurons versus non-neuronal cells; causal loci remain incompletely resolved (Garriga et al., 2018).
DNMT1-dependent hypermethylation	DRG neurons	Peripheral nerve injury	Kcna2/Kv1.2 promoter and 5′ UTR	Reduced potassium current, neuronal hyperexcitability, neuropathic pain	Strong locus-to-physiology evidence; demonstrates silencing of an excitability brake rather than a globally pro-pain role of methylation (Sun et al., 2019).
OCT1-DNMT3a axis	DRG neurons	Peripheral nerve injury	Kcna2 promoter	Selective Kcna2 repression, increased excitability, central sensitization	Strong causal evidence based on transcription-factor, enzyme, locus, electrophysiology, and behavioral linkage (Zhao et al., 2017).
Chromatin accessibility/enhancer remodeling	Bulk DRG and PNS tissue	CCI and CFA	Model-specific distal regulatory regions	Distinct injury- and inflammation-related transcriptional programs	Supports enhancer-level regulation, but cell composition and peak-to-gene assignment remain limitations (Stephens et al., 2021).
Nucleolin-dependent accessibility	DRG neurons	Peripheral nerve injury	Ccl2 promoter	CCL2 induction and persistent hypersensitivity	Recent locus-specific evidence that accessibility can directly activate a pro-nociceptive inflammatory gene (Zhang et al., 2026).
Global methylation/MeCP2 increase	Spinal cord, mixed cells	CCI	Multiple methylation-sensitive genes	5-azacytidine reduced hypersensitivity	Pharmacological and bulk-tissue evidence; target loci and responsible cell types are not fully resolved (Wang et al., 2011; Li et al., 2023a).
DNMT3a-mediated opioid-system repression	DRG and spinal pain pathways	Neuropathic pain models	MOR/OPRM1-related signaling	Reduced opioid receptor expression and impaired opioid analgesia	Links epigenetic repression to treatment response; regional and receptor-specific contributions require careful separation (Obara et al., 2009; Uchida et al., 2015; Shao et al., 2017; Sun et al., 2017).
Activity-regulated Dnmt3a2	Dorsal horn neurons	CFA inflammatory pain	Ptgs2/Cox-2 and activity-induced genes	Required for establishment of persistent hypersensitivity	Supports a memory-like consolidation mechanism, but does not imply that increased methylation is uniformly pro-nociceptive (Bayraktar and Kreutz, 2018; Oliveira et al., 2016; Oliveira et al., 2019).
Promoter demethylation	Spinal dorsal horn	SNL	Cxcr3/CXCL10 signaling	Enhanced excitatory transmission and neuropathic pain maintenance	Illustrates activation of a pro-nociceptive gene through loss of methylation (Jiang et al., 2017; Kong et al., 2021).
TET1-dependent hydroxymethylation	Dorsal horn neurons	SNL	Bdnf promoter	Increased BDNF and mechanical allodynia	Provides a second example in which demethylation-related activity is pro-nociceptive (Hsieh et al., 2016).
G9a/H3K9me2-mediated repression	DRG neurons	Nerve injury	Multiple K+ channel genes	Coordinated silencing of excitability brakes and transition to chronic pain	Strong genetic and pharmacological evidence for a shared repressive program across multiple channels (Laumet et al., 2015).
EZH2/H3K27me3 signaling	Spinal neurons and glia	Peripheral nerve injury	Neuroinflammatory pathways	EZH2 inhibition reduced cytokines, glial markers, and pain behavior	Demonstrates that a nominally repressive writer can promote pain indirectly by silencing anti-inflammatory or regulatory genes (Yadav and Weng, 2017).
Histone lysine crotonylation	Ganglion macrophages, sensory neurons, glia	Trigeminal and spinal neuropathic pain models	Inflammatory cytokine and chemokine programs	p300 inhibition reduced inflammation and pain behavior	Cell-type direction was heterogeneous, underscoring the need to avoid assigning one global function to a histone mark (Zou et al., 2022).
Longitudinal methylome reprogramming	Prefrontal cortex	SNI, 1 day to 1 year	Stage-dependent gene networks	Early stimulus-response and later immune-related signatures	Compelling temporal association, but causal links to pain memory and affective symptoms remain limited (Topham et al., 2020).
METTL14-m6A-IGF2BP2 axis	DRG neurons	Chemotherapy-induced neuropathic pain	Grin2a/GluN2A mRNA stability	Enhanced presynaptic NMDAR signaling and persistent hypersensitivity	Mechanistically strong epitranscriptomic evidence; should not be classified as chromatin remodeling (Lu et al., 2024).

## 3 Peripheral sensitization: repression of excitability brakes and activation of injury programs

The DRG is both a conduit and a regulatory compartment for nociceptive input. After nerve injury, sensory neurons undergo persistent changes in ion channels, receptors, neuropeptides, and inflammatory mediators, while satellite glial cells, macrophages, Schwann cells, fibroblasts, and vascular cells can modify the local transcriptional environment (Jang and Garraway, 2024; Krames, 2014). Bulk methylome studies demonstrate that nerve injury produces durable and widespread DNA methylation changes in the DRG, including substantial alterations outside CpG islands (Garriga et al., 2018). These data support a genome-wide response, but they do not by themselves establish whether hypomethylation is pathogenic, compensatory, or partly attributable to shifts in cell composition.

The clearest causal examples involve methylation-dependent repression of potassium-channel genes. Nerve injury increases DNMT1 in DRG neurons through CREB-dependent signaling, promotes methylation of the Kcna2 promoter and 5′ untranslated region, reduces Kv1.2 expression and potassium current, and increases neuronal excitability; DNMT1 knockdown reverses these changes and attenuates neuropathic pain (Sun et al., 2019). A parallel OCT1-DNMT3a pathway targets a defined Kcna2 promoter region and selectively represses Kcna2 rather than related channel genes (Zhao et al., 2017). Together with G9a-dependent H3K9me2 across multiple potassium-channel promoters, these studies identify a convergent principle: chronic pain can be stabilized by coordinated epigenetic silencing of genes that normally constrain membrane excitability (Laumet et al., 2015).

Complementary evidence indicates that peripheral nociceptor excitability is also shaped by ion-channel-associated membrane signaling outside the epigenetic layer. In a bone cancer pain model, Na+/K + -ATPase alpha1 (NKAalpha1) interacted with P2X3 receptors in DRG neurons; loss of NKAalpha1 in TRPV1-positive sensory neurons enhanced P2X3R-dependent Ca2+ influx and neuronal hyperexcitability, increased CCL5 release, and promoted downstream spinal glial activation and pain hypersensitivity (Huang et al., 2025). This pathway provides a mechanistic bridge between peripheral ion-channel regulation and neuroimmune amplification, while remaining conceptually distinct from chromatin-mediated regulation.

Chromatin accessibility provides a complementary mechanism. ATAC-seq and RNA-seq analyses show that nerve injury and inflammation generate partly distinct accessible chromatin landscapes, with many changes in intronic and intergenic regions rather than canonical promoters (Stephens et al., 2021). This observation shifts attention toward enhancers and distal regulatory elements, but peak-to-gene assignments remain inferential without chromatin-conformation or locus-perturbation experiments. Recent evidence that nucleolin increases accessibility at the Ccl2 promoter in injured DRG neurons and that nucleolin knockdown reduces CCL2 expression and pain behavior provides a more direct locus-specific example (Zhang et al., 2026). Human DRG transcriptomic and epigenomic maps further reveal species and sex differences that may influence translation of rodent mechanisms (Ray et al., 2018; Franco-Enzástiga et al., 2025).

Synthesis. Peripheral sensitization is therefore better organized around two recurrent regulatory operations than around individual studies: repression of anti-excitatory genes and activation of injury-responsive or inflammatory loci. Both operations can involve DNA methylation, histone modification, or accessibility changes. The therapeutic implication is not to globally increase or decrease methylation, but to restore specific regulatory programs in defined sensory-neuron or non-neuronal subtypes.

## 4 Central sensitization and spinal transcriptional plasticity

Central sensitization includes strengthened excitatory transmission, reduced inhibition, activity-dependent gene induction, and glial amplification in the spinal dorsal horn (Woolf, 2010; Costigan et al., 2009; Ji et al., 2018; Todd, , 2022; L et al., 2018). Early studies reported increased global DNA methylation and MeCP2 expression after CCI and analgesic effects of 5-azacytidine, while subsequent work identified several hypermethylated spinal genes involved in glutamatergic modulation, GABA synthesis, adrenergic signaling, and ion-channel function (Wang et al., 2011; Li et al., 2023a). These findings established involvement of DNA methylation, but broad inhibitor experiments cannot distinguish beneficial demethylation of anti-nociceptive genes from potentially harmful activation of pro-nociceptive genes.

Epigenetic repression can also weaken endogenous or pharmacological analgesia. Neuropathic pain is associated with reduced opioid receptor expression in peripheral and spinal pathways (Obara et al., 2009). DNMT3a-dependent methylation and related chromatin changes can reduce MOR/OPRM1 expression, whereas DNMT or HDAC inhibition can restore receptor expression or morphine responsiveness in animal models (Uchida et al., 2015; Shao et al., 2017; Sun et al., 2017). These data are clinically relevant because they connect disease-associated transcriptional state to treatment efficacy, but they do not establish that epigenetic therapy will improve opioid outcomes in humans or avoid tolerance, dependence, and off-target effects.

Activity-regulated Dnmt3a2 provides a mechanistic bridge between nociceptive plasticity and molecular memory. Dnmt3a2 participates in activity-dependent gene regulation and memory processes (Bayraktar and Kreutz, 2018; Oliveira et al., 2016). In CFA-induced inflammatory pain, dorsal-horn Dnmt3a2 increased, and knockdown reduced Ptgs2/Cox-2 induction and prevented long-lasting hypersensitivity (Oliveira et al., 2019). Related work shows that neuronal activation can recruit *de novo* methylation at enhancers, although such general neuronal findings should not be presented as direct pain evidence (Kameda et al., 2021). In bone cancer pain, DNMT3a-mediated silencing of Kv1.2 in the dorsal horn provides an additional example of locus-specific repression in a distinct pain context (Miao et al., 2017).

Recent multi-omic work provides convergent, non-epigenetic evidence that transcriptional loss of potassium-channel-mediated excitability brakes also occurs in the spinal cord. In CCI-induced neuropathic pain, Nono was downregulated together with Kcnq2 in spinal neurons; restoring Nono increased Kcnq2 expression and outward K+ currents and attenuated hypersensitivity, while promoter-enrichment and reporter assays supported direct transcriptional control of Kcnq2 (Chen et al., 2026). Although the Nono-Kcnq2 pathway is not itself an epigenetic mechanism, it reinforces the broader principle that persistent pain can be sustained by transcriptional suppression of ion-channel programs that normally constrain neuronal excitability.

The most important counterpoint is that pain-related DNA methylation is bidirectional. SNL-induced demethylation of the Cxcr3 promoter increases C/EBP binding, CXCR3 expression, excitatory transmission, and pain maintenance (Jiang et al., 2017; Kong et al., 2021). TET1-dependent conversion of 5-methylcytosine to 5-hydroxymethylcytosine at the Bdnf promoter similarly increases BDNF in dorsal-horn neurons (Hsieh et al., 2016). Thus, hypermethylation may silence anti-nociceptive genes, whereas demethylation may activate pro-excitatory genes. This bidirectionality directly argues against treating DNMT inhibition as a universal analgesic strategy.

Synthesis. The spinal evidence supports a regulatory-balance model: chronic pain persists when excitatory and inflammatory programs gain access to transcription while inhibitory, potassium-channel, and opioid programs are repressed. The strongest therapeutic rationale is therefore locus- and cell-specific correction, not global normalization of a single epigenetic mark.

## 5 Histone modifications at neuroimmune interfaces

Neuroinflammation emerges from interactions among nociceptive neurons, microglia, astrocytes, satellite glia, infiltrating macrophages, and other immune cells. Histone modifications can regulate this interaction by changing the accessibility and transcriptional competence of cytokine, chemokine, inhibitory, and metabolic genes (Ji et al., 2018; Jiang et al., 2023; Wang et al., 2018; Penas and Navarro, 2018). Recent work also illustrates how peripheral excitability can feed into this interface: disruption of NKAalpha1-P2X3R regulation in TRPV1-positive DRG neurons increased CCL5 release and spinal glial activation in bone cancer pain (Huang et al., 2025). A critical distinction is that neither acetylation nor methylation has an invariant pro- or anti-inflammatory meaning; the effect depends on which locus is modified and in which cell.

Histone acetylation is generally associated with increased transcriptional competence. At pro-inflammatory loci, increased acetylation can facilitate NF-κB, AP-1, STAT3, or IRF-family activity and promote mediators such as CCL2, CX3CL1, TNF-α, and IL-1β (Jiang et al., 2023; Wang et al., 2018). Conversely, HDAC inhibition can increase expression of inhibitory or anti-inflammatory genes and reduce pain in some models, including restoration of GABAergic or opioid-related programs (Uchida et al., 2015; Wang et al., 2018; Bai et al., 2010). These apparently opposing observations are not contradictory: acetylation amplifies the regulatory program of the targeted gene. Consequently, enzyme class alone is an inadequate guide to therapeutic direction.

Histone methylation further illustrates context dependence. G9a-dependent H3K9me2 represses a broad set of potassium-channel genes in injured DRG neurons and is required for the acute-to-chronic transition in neuropathic pain (Laumet et al., 2015). By contrast, EZH2, which catalyzes H3K27 methylation, promotes spinal neuroinflammation and pain in part through repression of regulatory programs; pharmacological inhibition reduces glial markers, cytokines, and hypersensitivity (Yadav and Weng, 2017). The H3K27 demethylase JMJD3/KDM6B can also support inflammatory gene activation and macrophage infiltration in a lumbar spinal stenosis model (Lee et al., 2021). The coexistence of pro-pain effects from both a methyltransferase and a demethylase is a reminder that target genes and cell states, rather than the net amount of histone methylation, determine function.

Histone lysine crotonylation is an emerging example. Peripheral nerve injury altered crotonylation in a cell-type-dependent manner: levels increased in macrophages and microglia but decreased in some sensory neurons. Inhibition of p300 with C646 reduced inflammatory mediators and pain behavior in trigeminal and spinal models (Zou et al., 2022). These results are mechanistically interesting but remain preclinical, and C646 cannot be assumed to act exclusively through crotonylation rather than other p300-dependent acylation pathways.

Synthesis. The histone literature should be interpreted as evidence for locus-specific neuroimmune state transitions, not as evidence that one global histone mark is intrinsically nociceptive. Future studies need cell-resolved profiling before and after pain onset, coupled to perturbation of defined enhancers or promoters, to distinguish initiating changes from downstream inflammatory consequences.

## 6 Supraspinal chromatin dynamics, pain memory, and comorbidity

Chronic pain recruits prefrontal, cingulate, amygdalar, hippocampal, thalamic, hypothalamic, periaqueductal, and mesolimbic circuits that influence salience, avoidance, reward, mood, and cognition (Yang and Chang, 2019; Baliki Marwan and Apkarian, 2015). Longitudinal methylome analysis after SNI showed that prefrontal regulatory changes evolve from early stimulus-response programs toward later immune-related signatures, while cytoskeletal pathways remain involved over extended periods (Topham et al., 2020). This temporal structure is more informative than a static list of differentially methylated genes because it suggests that the molecular state accompanying pain chronification changes with disease stage.

Brain-region specificity is equally important. After neuropathic injury, TET and DNMT-family expression can change in opposite directions across the medial prefrontal cortex, amygdala, caudate-putamen, and thalamus (Rodrigues et al., 2023). Histone marks associated with active transcription were reduced in the periaqueductal gray, lateral hypothalamus, and nucleus accumbens but not uniformly in the ventral tegmental area (Bryant et al., 2022). These findings indicate that the chronic-pain brain does not enter one homogeneous epigenetic state.

The concept of “pain memory” is plausible because durable chromatin states regulate memory allocation and engram stability in other learning paradigms (Oliveira et al., 2016; Santoni et al., 2024). However, most pain studies have not yet demonstrated that a specific epigenetic change in an identified neuronal ensemble is necessary and sufficient for persistent pain memory or affective comorbidity. Region-level methylation or histone associations should therefore be described as candidate mechanisms of long-term circuit adaptation rather than direct proof that pain is stored as an epigenetic memory.

Synthesis. Supraspinal evidence is strongest for temporal and regional heterogeneity, but weakest for direct causal attribution to pain memory. Progress will require ensemble- or cell-type-specific perturbation combined with behavioral separation of sensory, affective, motivational, and cognitive dimensions.

## 7 Non-coding RNA-chromatin crosstalk and RNA m6A epitranscriptomics

### 7.1 Non-coding RNAs at the chromatin interface

miRNAs, lncRNAs, and circRNAs are extensively dysregulated in injured DRG, spinal cord, and patient-derived samples and can regulate inflammatory mediators, ion channels, receptors, axonal responses, and glial activation (Jiang M. et al., 2022; He et al., 2024; Zhou et al., 2017; Li et al., 2023b; Felix et al., 2022). Most of these actions are post-transcriptional and should not automatically be labelled chromatin remodeling. The chromatin-associated subset comprises ncRNAs that recruit or exclude DNA methyltransferases, histone modifiers, transcriptional repressors, or ATP-dependent remodeling complexes, or that alter transcription through antisense interactions at a genomic locus.

The miR-30c-5p-DNMT axis illustrates multilayered crosstalk. Manipulating miR-30c-5p alters DNMT3a/3b expression and methylation states at genes including TGF-β1 and the miR-30c host locus, producing sustained antinociceptive effects in rodents (Tramullas et al., 2018; Francés et al., 2022). A nerve-injury-specific lncRNA that increases Ccl2 expression provides another example of a transcriptionally relevant ncRNA pathway (Du et al., 2022). More generally, reciprocal regulation between ncRNAs and SWI/SNF, ISWI, CHD, and INO80 complexes is biologically plausible, but direct pain-specific evidence for many proposed interactions remains sparse (Bure and Nemtsova, 2023).

### 7.2 RNA m6A as a distinct epitranscriptomic mechanism

RNA m6A is deposited by writer complexes including METTL3, METTL14, and WTAP, removed by erasers such as FTO and ALKBH5, and interpreted by reader proteins including YTH-domain and IGF2BP-family proteins. These processes influence RNA processing, stability, localization, and translation rather than nucleosome organization (Lu et al., 2024; Zhang L. et al., 2022; Zhang K. et al., 2022). In chemotherapy-induced neuropathic pain, METTL14-dependent m6A modification of Grin2a mRNA and IGF2BP2 binding increase transcript stability, GluN2A expression, presynaptic NMDAR activity, and hypersensitivity (Lu et al., 2024). METTL3-dependent regulation of primary miR-150 processing provides a second example of interaction between epitranscriptomics and ncRNA biology (Zhang L. et al., 2022).

Synthesis. ncRNAs and m6A should be retained in the review only within a clearly labelled integrative framework. ncRNAs may participate in chromatin regulation, post-transcriptional regulation, or both; m6A is epitranscriptomic. Their inclusion expands the regulatory network relevant to chronic pain but should not erase mechanistic boundaries.

## 8 Human evidence and biomarker candidates

Human evidence remains substantially less mature than the mechanistic animal literature. Peripheral-blood methylation studies have reported differences between nociceptive and neuropathic pain, associations between TRPA1 promoter methylation and DN4 neuropathic features, and methylation signatures related to conditioned pain modulation in chronic low back pain (Stenz et al., 2021; Takenaka et al., 2020; Goodin et al., 2022). CRPS, FKBP5-related signatures, and painful diabetic neuropathy have also yielded candidate loci or genome-wide patterns (Bruehl et al., 2019; Maiarú et al., 2023; Kwiatkowska et al., 2025). These studies demonstrate measurable association, not clinical validity Figure 1.

**FIGURE 1:**
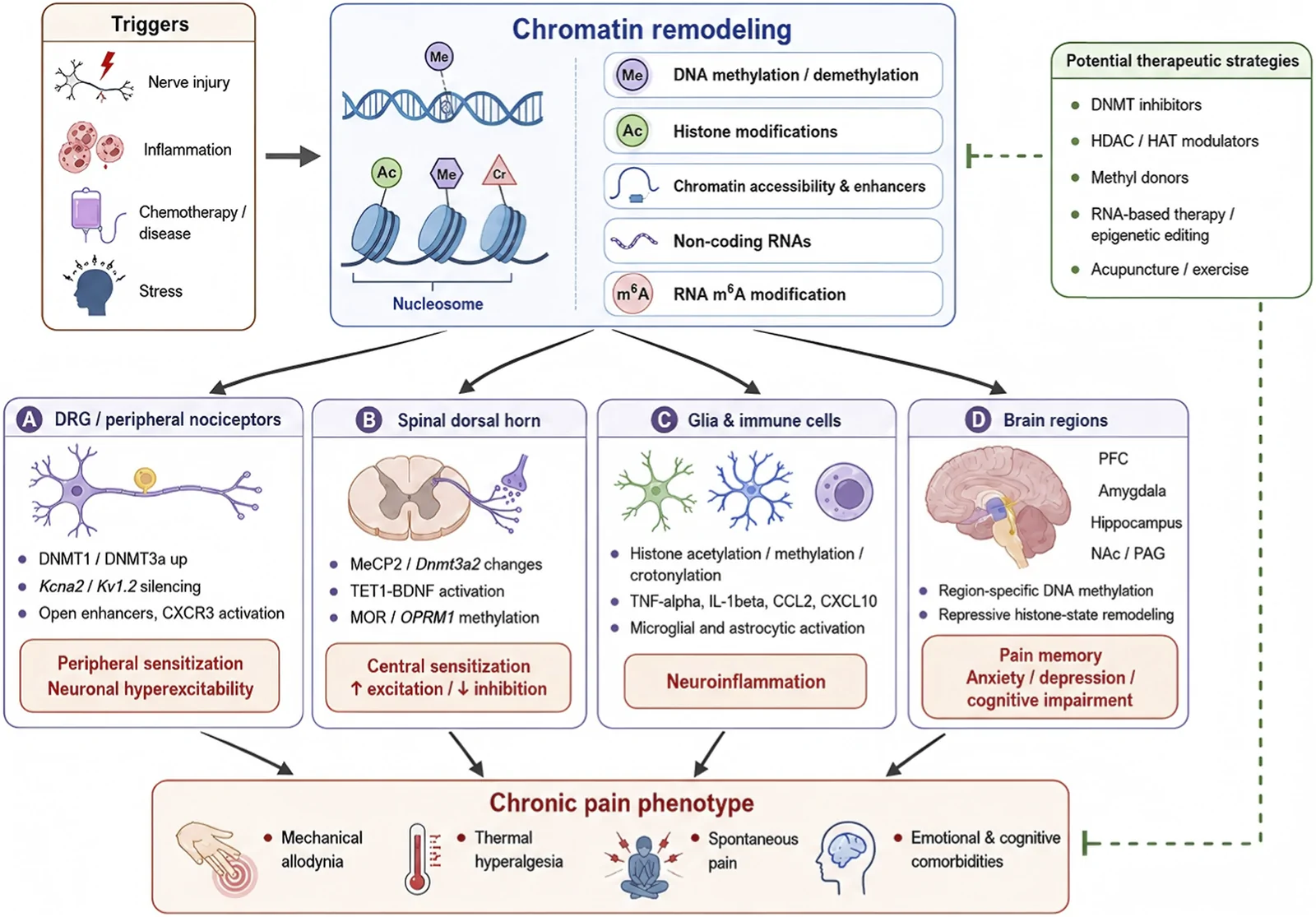
Conceptual framework linking regulatory layers to chronic pain. The four lower panels show **(A)** dorsal root ganglia (DRG)/peripheral nociceptors, **(B)** the spinal dorsal horn, **(C)** glial and immune cells, and **(D)** brain regions. DNA methylation and hydroxymethylation, histone modifications, and chromatin accessibility are chromatin-associated epigenetic mechanisms. Non-coding RNAs may act at chromatin or post-transcriptionally. RNA m6A is shown as an interacting but conceptually distinct epitranscriptomic layer. Injury, inflammation, disease, and stress can engage these mechanisms in peripheral sensory ganglia, the spinal dorsal horn, glial and immune cells, and higher brain regions, contributing to peripheral sensitization, central sensitization, neuroinflammation, and pain-related affective or cognitive changes. Therapeutic strategies are presented as investigational because most evidence is preclinical and broad epigenetic interventions lack cell and locus specificity.

Three limitations should moderate biomarker claims. First, peripheral blood is a heterogeneous tissue whose methylation profile is strongly influenced by leukocyte composition, age, sex, smoking, medication, metabolic disease, and stress. Second, a blood signature need not reproduce the regulatory state of DRG, spinal cord, or brain. Overlap between T-cell and prefrontal methylation after SNI is an informative translational bridge, but it remains an animal observation (Massart et al., 2016). Third, most studies are cross-sectional, modest in size, and lack independent external validation, prespecified thresholds, or demonstration that the biomarker changes diagnosis, prognosis, or treatment selection.

Accordingly, the appropriate terms are “candidate biomarker” and “biomarker potential,” not “diagnostic biomarker” or “clinically validated signature.” Table 2 separates evidence source from validation status and highlights the next steps needed for clinical utility.

**TABLE 2:** Candidate epigenetic biomarkers and current validation status.

Candidate/signature	Pain context	Sample source	Reported association	Current evidence level	Main validation gap
Genome-wide methylation profiles	Nociceptive versus neuropathic chronic pain	Human peripheral blood	Different pathway-enriched methylation patterns between clinical subtypes	Exploratory human association	Independent multicenter replication, adjustment for blood-cell composition, and classification performance in prospective cohorts (Stenz et al., 2021).
TRPA1 promoter CpG −51	Neuropathic pain features	Human peripheral blood	Methylation correlated with DN4 score	Single-cohort candidate	Specificity for neuropathic pain, stability over time, treatment responsiveness, and external validation (Takenaka et al., 2020).
OPRM1/ADRB2/CACNA2D3-related profiles	Chronic low back pain/conditioned pain modulation	Human peripheral blood	Differential methylation associated with CPM phenotype	Exploratory epigenome-wide association	Replication, causal interpretation, standardized CPM protocols, and control of confounding (Goodin et al., 2022).
Overlapping PFC and T-cell methylation	Persistent neuropathic pain	Rodent PFC and peripheral T cells	Partial overlap; T-cell sites distinguished SNI from sham animals	Preclinical translational bridge	Human confirmation and demonstration that peripheral signals track central pain mechanisms (Massart et al., 2016).
COL11A1/HLA-related methylation	Post-traumatic CRPS	Human blood	Differentially methylated CpGs enriched in immune pathways	Exploratory human association	Larger cohorts, comparison with trauma controls, and prospective risk prediction (Bruehl et al., 2019).
FKBP5 methylation signature	Chronic pain with neuropathic component	Human blood	Association with neuropathic features	Candidate mechanistic biomarker	External replication, tissue specificity, and incremental clinical utility beyond questionnaires (Maiarú et al., 2023).
Whole-genome methylation signatures	Painful diabetic neuropathy	Human blood	Distinct genome-wide methylation patterns	Recent human discovery study	Prospective validation, diabetic controls without pain, medication effects, and prediction of treatment response (Kwiatkowska et al., 2025).

## 9 Therapeutic strategies and translational readiness

The reversibility of epigenetic regulation is attractive therapeutically, but reversibility does not equal druggability (Luo et al., 2021). Most available inhibitors act broadly across tissues and genomic loci, whereas pain mechanisms are cell-, locus-, and stage-specific. Therapeutic claims should therefore be calibrated to evidence level and separated into preclinical proof of mechanism, human safety/efficacy testing, and clinically validated use.

### 9.1 DNMT inhibitors

5-Azacytidine, 5-aza-2′-deoxycytidine, and RG108 reduce hypersensitivity and restore selected genes in animal models (Wang et al., 2011; Li et al., 2023a; Shao et al., 2017). However, nucleoside DNMT inhibitors were developed primarily for proliferative disorders, can produce widespread demethylation and toxicity, and may activate pro-nociceptive genes such as Cxcr3 or Bdnf-related pathways. At present, these compounds are better regarded as mechanistic probes than as near-term analgesics.

### 9.2 HDAC and HAT modulators

Broad or isoform-selective HDAC inhibition can increase anti-inflammatory, inhibitory, or opioid-related gene expression and reduce pain behavior in rodents (Uchida et al., 2015; Wang et al., 2018; Bai et al., 2010; Westlund et al., 2023). Yet isoform, cellular compartment, dose, and disease stage may determine whether the net effect is beneficial. Most importantly, a 12-week phase two randomized trial in 282 participants with painful diabetic peripheral neuropathy found that the HDAC6 inhibitor ricolinostat was not superior to placebo for pain or neuropathy outcomes (Michelson et al., 2023). This negative trial should temper claims that preclinical HDAC6 biology has already translated into analgesic efficacy. The p300 inhibitor C646 reduces crotonylation-associated inflammation in rodents, but its specificity and human safety for chronic pain are unknown (Zou et al., 2022).

### 9.3 Histone methyltransferases and demethylases

G9a and EZH2 inhibition and JMJD3 blockade show analgesic or anti-inflammatory effects in animal models (Laumet et al., 2015; Yadav and Weng, 2017; Lee et al., 2021). These targets may offer more pathway resolution than global DNMT inhibition, but they remain involved in development, immunity, cancer biology, and broad transcriptional control. Cell-restricted delivery and a defined therapeutic window will be essential before clinical testing.

### 9.4 Methyl donors and metabolic interventions

DNA and histone methylation depend on one-carbon metabolism and S-adenosylmethionine (SAM) availability (Yang et al., 2020; Mentch and Locasale, 2015). Prolonged SAM treatment partially reversed nerve-injury-associated methylation signatures in mouse frontal cortex and reduced pain behavior (Topham et al., 2021). Because methylation changes are bidirectional, metabolic supplementation should not be assumed to normalize the epigenome globally; dose, timing, tissue exposure, and long-term metabolic consequences require clarification.

### 9.5 RNA-directed therapy and locus-specific epigenome editing

Antisense oligonucleotides, miRNA modulators, RNA-targeting systems, and dCas9-based epigenome editing could in principle act at defined transcripts or genomic loci. Their conceptual advantage is precision, but delivery to DRG neurons, spinal cell types, or specific brain ensembles remains a major barrier. Off-target transcriptional effects, immunogenicity, reversibility, and durability must be measured in pain-relevant human models before therapeutic claims are justified.

### 9.6 Non-pharmacological interventions

Acupuncture, exercise, sleep and circadian interventions, nutrition, psychological therapies, and other non-pharmacological approaches may alter stress, immune, metabolic, and neuronal signaling that secondarily influences epigenetic state. In a mouse neuropathic-pain model, prolonged Table 3 acupuncture improved sensory and neurobehavioral outcomes and altered prefrontal DNA methylation-related markers (Jang et al., 2020). These findings are hypothesis-generating; epigenetic change may be a mediator, consequence, or correlate of improved behavior and should not be described as a proven mechanism in patients.

**TABLE 3:** Therapeutic evidence separated by preclinical and clinical status.

Intervention	Proposed regulatory target	Evidence in pain	Human status	Key limitation	Translational interpretation
5-azacytidine/5-aza-2′-deoxycytidine	DNMT inhibition/global demethylation	Analgesic effects in rodent neuropathic pain	No established chronic-pain indication	Broad genomic effects, toxicity, poor cell specificity, possible activation of pro-pain loci	Mechanistic tools rather than near-term analgesics (Wang et al., 2011; Li et al., 2023a).
RG108	Non-nucleoside DNMT inhibition	Restored MOR-related expression and reduced hypersensitivity in rodents	No validated pain efficacy	Limited human pharmacology and locus specificity	Useful proof of principle; requires targeted delivery (Shao et al., 2017).
TSA/VPA/SAHA/FK228 and related HDAC inhibitors	Histone acetylation	Analgesic and opioid-sensitizing effects in multiple animal models	No class-wide clinical validation for chronic pain	Isoform- and cell-dependent effects; systemic toxicity	Preclinical signal does not justify broad therapeutic claims (Uchida et al., 2015; Wang et al., 2018; Bai et al., 2010).
Ricolinostat	HDAC6 inhibition	Strong preclinical rationale	Phase 2 randomized trial negative in painful DPN	No superiority to placebo over 12 weeks	Direct evidence of a preclinical-to-clinical translation gap (Michelson et al., 2023).
G9a inhibition/conditional deletion	H3K9 methylation	Restored K+ channel programs and prevented chronic pain in rodents	No pain trial	Essential functions beyond nociceptive neurons	Promising causal target; delivery and safety unresolved (Laumet et al., 2015).
DZNep/GSK126	EZH2/PRC2 signaling	Reduced glial activation, cytokines, and pain behavior	No pain trial	Broad PRC2 effects, cancer/immune relevance	Preclinical target requiring cell-specific validation (Yadav and Weng, 2017).
GSK-J4-like JMJD3 inhibition	H3K27 demethylation	Reduced macrophage infiltration and pain in LSS model	No pain trial	Model-specific evidence and broad immune effects	Early preclinical strategy (Lee et al., 2021).
C646	p300-dependent acetylation/crotonylation	Reduced inflammatory transcription and pain behavior	No pain trial	Target specificity and pharmacokinetics	Mechanistic probe for metabolism-chromatin-inflammation crosstalk (Zou et al., 2022).
SAM supplementation	Methyl-donor metabolism	Partial reversal of cortical DMRs and behavioral improvement in mice	No validated analgesic indication	Bidirectional methylation, systemic metabolic effects	Requires dose, tissue, and long-term safety studies (Yang et al., 2020; Mentch and Locasale, 2015; Topham et al., 2021).
Acupuncture	Indirect neuroimmune/metabolic regulation	Epigenetic and behavioral changes in mouse PFC	Clinical analgesic literature exists, but epigenetic mediation unvalidated	Causality and protocol heterogeneity	Do not equate epigenetic association with proven clinical mechanism (Jang et al., 2020).
dCas9 epigenome editing/RNA therapeutics	Locus- or transcript-specific regulation	Conceptual and early experimental rationale	No established chronic-pain therapy	Delivery, off-target effects, durability, immunogenicity	Most aligned with locus specificity, but technologically immature.

## 10 Challenges and research priorities

First, cellular resolution remains inadequate. DRG neurons, satellite glia, Schwann cells, macrophages, spinal neurons, microglia, astrocytes, and lymphocytes may exhibit opposing changes that are averaged in bulk tissue. Single-cell or single-nucleus methylome, ATAC-seq, CUT&Tag, and transcriptomic approaches should be paired with cell-type-specific perturbation rather than used solely as descriptive atlases.

Second, time must be treated as a mechanistic variable. Regulatory states during injury initiation, consolidation, maintenance, remission, and relapse may differ. A target that prevents chronification may not reverse established pain, and an intervention that is beneficial in one stage may disrupt compensatory programs in another. Longitudinal designs and prespecified temporal windows are therefore essential.

Third, association must be separated from causation. Demonstrating a changed mark near a pain-related gene is insufficient. Strong causal studies should show that the alteration precedes or maintains pain, changes target-gene transcription in the relevant cell, affects physiology or circuit function, and produces a behavioral effect that can be rescued by locus-specific correction.

Fourth, sex, species, and tissue context require explicit consideration. Human DRG chromatin accessibility differs by sex even in donors without chronic pain (Franco-Enzástiga et al., 2025), and rodent-to-human conservation cannot be assumed. Future studies should integrate human DRG, spinal tissue when available, induced sensory neurons, organoids, and clinically annotated peripheral samples.

Fifth, biomarker development needs a clinical-use framework. Studies should define the intended use—diagnosis, prognosis, treatment selection, or pharmacodynamic monitoring—before discovery; control cell composition and major confounders; use independent validation cohorts; report discrimination and calibration; and test whether the marker improves decisions beyond standard clinical variables.

Finally, therapeutic development should prioritize precision and falsifiability. Candidate interventions should specify the target cell, locus or transcript, disease stage, delivery route, expected on-target biomarker, and safety boundary. Negative studies, including the ricolinostat trial, are particularly valuable because they prevent premature extrapolation from molecular plausibility to clinical efficacy.

## 11 Conclusion

Chronic pain is associated with durable regulatory changes across peripheral, spinal, immune, and supraspinal systems, but these changes do not form a single uniform “pain epigenome.” The most robust principle is context dependence: hypermethylation can silence anti-nociceptive genes, demethylation can activate pro-nociceptive genes, and the same class of histone modification can support inflammatory or inhibitory transcription according to locus and cell type. ncRNAs occupy both chromatin-associated and post-transcriptional roles, whereas RNA m6A is an epitranscriptomic mechanism that should remain conceptually distinct.

The field has moved beyond simple correlation in several well-defined pathways, including DNMT1/DNMT3a-Kcna2, G9a-dependent potassium-channel repression, TET1-Bdnf, and METTL14-m6A-IGF2BP2-Grin2a. Nevertheless, most therapeutic and biomarker evidence remains preclinical or exploratory. Broad epigenetic inhibitors should not be presented as disease-modifying treatments without cell- and locus-specific validation, and peripheral methylation signatures should not be described as clinically established biomarkers. The most credible path toward translation combines longitudinal cell-resolved profiling, causal epigenome or RNA perturbation, validation in human pain-relevant tissues, and prospective clinical studies designed to demonstrate practical utility.
